# Perilla Seed Hulls Polyphenol Extract: Molecular Characterization and Suppression of LPS‐Induced Inflammation in RAW 264.7 Cells via NF‐κB Signaling Pathway

**DOI:** 10.1002/fsn3.70847

**Published:** 2025-09-03

**Authors:** Di Xie, Bingcong Wang, Yanrong Zhang, Yinlu Gao

**Affiliations:** ^1^ College of Food Science and Engineering Jilin Agricultural University Changchun China; ^2^ College of Chemistry Baicheng Normal University Baicheng China; ^3^ School of Clinical Medicine Baicheng Medical College Baicheng China; ^4^ School of Biological and Food Engineering Jilin Engineering Normal University Changchun China; ^5^ Jilin Provincial Collaborative Innovation Center for Coarse Cereals Resources Development and Industrialization Jilin Engineering Normal University Changchun China; ^6^ Jilin‐Xinjiang Joint Laboratory for Scientific and Technological Innovation in Medicine‐Food Homology Industry Jilin Engineering Normal University Changchun China

**Keywords:** anti‐inflammatory, NF‐κB, perilla, polyphenol, seed hulls

## Abstract

As a dual‐purpose medicinal and edible resource, Perilla seeds are rich in bioactive compounds. There are very few studies on the by‐product of perilla seed hells. Moreover, there is a lack of systematic research on the chemical composition and biological activity of perilla seed hell polyphenols. We employed ultrasound‐assisted extraction (60% ethanol, 410 W power, 30 min, liquid–solid ratio 30:1 mL/g) to obtain polyphenols from PSH, yielding perilla seed hulls polyphenol crude extraction (PSH‐C), and further verified the presence of polyphenolic compounds using Fourier transform infrared spectroscopy (FT‐IR). Subsequently, PSH‐C was further separated and purified using HPD600 macroporous resin to obtain a purified perilla seed hulls polyphenol extract (PSH‐P). The polyphenolic composition of PSH‐P was characterized by UPLC‐MS/MS, and the major polyphenols were quantitatively analyzed using authentic standards. Antioxidant assays demonstrated that PSH‐P could effectively scavenge free radicals. PSH‐P significantly inhibited nitric oxide (NO) production, suppressed the production of inflammatory factors, and downregulated the transcription of iNOS and COX‐2. PSH‐P likely exerts anti‐inflammatory effects by suppressing LPS‐induced nuclear translocation of NF‐κB (p65) and inhibiting the expression of P‐p65 and P‐IκBα, thereby blocking NF‐κB signaling pathway activation. PSH‐P likely exerts its anti‐inflammatory effects by blocking NF‐κB pathway activation through suppression of LPS‐induced nuclear translocation of p65 and inhibition of P‐p65 and P‐IκBα expression.

## Graphical abstract

This study employed UPLC‐MS/MS and UPLC for the qualitative and quantitative analysis of polyphenols from Perilla seed shells. The anti‐inflammatory activity of these polyphenols was evaluated, and their inhibitory mechanism on inflammation via the NF‐κB signaling pathway was explored.

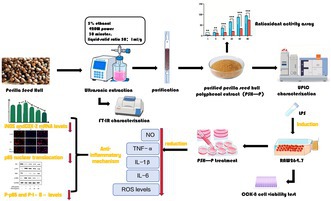

## 1 Introduction

Perilla is an annual herbaceous plant in the genus Perilla (Lamiaceae). It is widely distributed across regions including China, Russia, South Korea, Japan, Myanmar, and India (Li et al. 2018; Rouphael et al. 2019). Perilla is among the first batch of 60 dual‐purpose medicinal and edible items officially recognized by the Chinese Ministry of Health. It contains various biologically active chemical components, demonstrating unique medicinal and culinary value. Notably, its stems, leaves, and seeds are all officially listed in the Chinese Pharmacopeia (Li et al. 2018, 2015; Sun‐waterhouse et al. 2024; Uemura et al. 2018). Perilla seed hulls, a by‐product generated during *Perilla frutescens* oil extraction and seasoning production, are notably rich in diverse polyphenolic compounds, including apigenin, luteolin, and rosmarinic acid, etc. (Li et al. 2019; Sumneang et al. 2024). And substances such as cellulose. However, in conventional processing practices, this bioactive‐rich material is typically repurposed as animal feed or directly discarded as industrial processing waste. This practice not only contributes to environmental contamination but also represents significant resource wastage.

In recent years, growing health consciousness has driven cross‐disciplinary interest in polyphenols–naturally occurring bioactive compounds ubiquitously present in vegetables, fruits, cereals, and other plant sources. Emerging research has demonstrated their multifunctional properties, particularly antioxidant, anti‐inflammatory, anticancer, hypoglycemic, and hypolipidemic activities, which underpin their expanding applications in functional foods and preventive medicine (Shen et al. 2017; Nguyen et al. 2025; Chen et al. 2017; Yang et al. 2025; Tabussum et al. 2013). As a fundamental biological process, inflammation is initiated by pathogenic factors such as tissue trauma, infectious agents, and oxidative stress‐induced damage (Macías‐Cortés et al. 2022). The anti‐inflammatory effects of polyphenols stem from their capacity to suppress the secretion of multiple inflammatory mediators and further inhibit the triggering of associated signaling pathways, while concurrently mitigating oxidative damage through free radical scavenging in biological systems, thereby exhibiting potent antioxidant activity (Crascì et al. 2018; Chen et al. 2017). Compared to other botanical sources, polyphenolic compounds in *Perilla frutescens* seeds remain relatively understudied, with particularly scarce reports on their processing by‐product—perilla seed hulls. The polyphenolic constituents in the perilla seed hulls remain incompletely characterized, and their anti‐inflammatory activity has not been previously reported. Therefore, in this study, we plan to extract polyphenols from Perilla seed hulls using ultrasonic‐assisted ethanol extraction. This method will be employed due to its high efficiency, mild conditions, environmental friendliness, and capacity for enhanced preservation of bioactive compounds (Motikar et al. 2020). We will characterize the polyphenolic composition of PSH‐P by UPLC‐ESI‐MS/MS and quantify its major constituents by UPLC. Additionally, we will evaluate the in vitro antioxidant activity, anti‐inflammatory activity, and potential mechanism of action of PSH‐P.

## 2 Materials and Methods

### 2.1 Materials and Chemicals

Perilla seed hulls were supplied from Antu Fengyuda (Yanbian, China). The perilla seed hulls were dehydrated at 40°C for 12 h until constant mass was achieved. The perilla seed hulls were pulverized using a grinder, subsequently sieved through an 80‐mesh sieve.

Folin–Ciocalteu reagent (R033317) and vitamin C (VC; R017968) were purchased from Ron Chemical Co. Ltd. (Shanghai, China). Assay kits for 1,1‐Diphenyl‐2‐picrylhydrazyl (DPPH; A153‐1‐1) and 2,2′‐azino‐bis(3‐ethylbenzothiazoline‐6‐sulfonic acid) (ABTS; A015‐2‐1) were supplied by Nanjing Jiancheng Bioengineering Institute (Nanjing, China). All HPLC‐grade reference standards were obtained from Yuanye Bio‐Technology (Shanghai, China). The other reagents are either commercially available or produced in China. Among these, all reagents used in the mobile phase are of chromatographic grade, while the others are of analytical grade.

RAW264.7 cells were obtained from the Shanghai Cell Bank of the Chinese Academy of Sciences (catalog no. SCSP‐5036; China). Dulbecco's Modified Eagle Medium (DMEM; C3060) was purchased from VivaCell Biosciences (China). Fetal bovine serum (FBS; FSP500) was supplied by ExCell Biotech Co. Ltd. (Uruguay). Lipopolysaccharide (LPS; ST1470), the Cell Counting Kit‐8 (CCK‐8; C0037), ELISA kits for nitric oxide (NO; S0021S), BCA Protein Assay Kit (P0010S), and DAPI Staining Solution (C1006) were purchased from Beyotime Biotechnology Co. Ltd. (Shanghai, China). ROS Assay Kit (CA1410), TriQuick Reagent (R1100), and immunofluorescence reagents/antibodies were obtained from Solarbio Biotechnology (Shanghai, China). ELISA kits for tumor necrosis factor‐α (TNF‐α; LDQB‐20852), interleukin‐6 (IL‐6; LDQB‐20188), and interleukin‐1β (IL‐1β; LDQB‐20174) were supplied by Leda Qibo Biotechnology Co. Ltd. (Fujian, China).

### 2.2 Extraction and Purification of Polyphenols From PSH

The PSH sample was defatted with n‐hexane through three successive extractions, followed by centrifugation (5000 rpm, 15 min). Samples were dried to constant weight at 45°C and stored for subsequent use.

A defatted sample (10 g) was added to 300 mL of 60% ethanol solution and ultrasonicated for 30 min (410 W). The crude polyphenol extract from perilla seed husk (PSH‐C) was obtained by collecting the supernatant after centrifugation (12,000 rpm, 10 min) followed by freeze‐drying. The purification process employed HPD600 macroporous resin for PCH‐C, utilizing dynamic adsorption and desorption techniques to obtain PSH‐P. The methods for extraction and purification have been provided in the Appendix S1.

### 2.3 FT‐IR Analysis of PSH‐C

The sample and potassium bromide (KBr) were ground uniformly at a ratio of 1:150 (w/w). The spectral changes were measured by FT‐IR in the 4000–400 cm^−1^ frequency range. (Nicolet iS5, Thermo Fisher, USA).

### 2.4 Quantification of Polyphenol Content

Total phenolic content (TPC) was determined according to the method of Hooshmand et al. (2015) with minor modifications. The procedure involved combining 50 μL of test sample or gallic acid standard with 200 μL deionized water and 250 μL Folin–Ciocalteu reagent (diluted 1:8 in ddH_2_O). Following 6 min equilibration at ambient temperature, 250 μL 10% Na_2_CO_3_ solution was introduced. The mixture underwent 60 min incubation at 30°C under light‐protected conditions. Absorbance readings at 765 nm were acquired using a microplate reader (Bio‐Rad iMark, USA), with TPC calculated as gallic acid equivalents per gram extract (mg GAE/g E).

Total flavonoid content (TFC) was determined following the procedure of Macías‐Cortés et al. (2022) with minor modifications. A 500 μL aliquot of sample or rutin standard was reacted with 2 mL ddH_2_O and 150 μL 5% NaNO_2_ solution. After 6 min incubation at ambient temperature, 150 μL 10% AlCl_3_ solution was introduced, and the mixture was re‐incubated under identical conditions for 6 min. The reaction was quenched with 1 mL NaOH (1 M), and absorbance recorded at 510 nm. TFC values were normalized to rutin equivalents per gram extract (mg RE/g E).

### 2.5 UPLC MS/MS of Polyphenol

50 mg of PSH‐P sample was weighed, homogenized with 1500 μL of 70% methanol solution with internal standard, sonicated (360 W, 40 kHz) for 10 min, centrifuged (12,000 rpm) for 5 min, and the supernatant was then analyzed.

UPLC‐MS/MS analysis was performed following the method described by Chen et al. (2013) with minor modifications.

The composition of PSH‐P was analyzed using the UPLC‐ESI‐MS/MS system (ExionLC AD, USA). The parameters were set as follows: column, SB‐C18 (Agilent, 2.1 × 100 mm, 1.8 μm); mobile phase, solvent A (0.1% formic acid in water) and solvent B (0.1% formic acid in acetonitrile). Separation used a gradient from 95% A/5% B to 5% A/95% B (0–9 min); 5% A/95% B (9–10 min); 95% A/5% B (10–11.1 min), column stabilization at initial conditions (11.1–14 min). The system operated at a 0.35 mL/min flow rate, a 2 μL injection volume, and a 40°C column oven temperature. The LC effluent was alternately directed to an ESI‐QTRAP‐MS.

Electrospray ionization (ESI) conditions were configured as follows: 5500 V (positive mode)/−4500 V (negative mode), source temperature 500°C; ion spray voltage; GSI 50 psi; GSII 60 psi; CUR 25 psi. QQQ‐MS operated in MRM mode for all analyses, with relevant settings optimized for target analytes.

Using the MWDB database (Metware Biotechnology, China), MS/MS spectrometry data was qualitatively analyzed. To minimize experimental interference, isotope signals, duplicate ions of NH^4+^, Na^+^, K^+^, as well as fragment ions of larger molecules were removed.

### 2.6 Quantification of Phenolic Compounds

Dissolve 1.00 g of PSH‐P in 100 mL of 70% aqueous methanol. Vortex‐mix the solution for 30 s and ultrasonicate for 10 min (360 W power, 40 kHz frequency). Following centrifugation (12,000 rpm, 3 min), 1 mL of supernatant was precisely aliquoted, subjected to a 10‐fold dilution, membrane‐filtered (0.22 μm), and analyzed by HPLC.

PSH‐P's primary phenolics were quantified via ACQUITY UPLC H‐Class PLUS with PDA detection (Waters, USA). The parameters were set as follows: column ACQUITY Premier BEH C18 (Waters, 2.1 × 50 mm, 1.7 μm), temperature, 35°C, mobile phase, solvent A (0.1% formic acid in water) and solvent B (acetonitrile). Separation used a gradient from 95% A/5% B (0 min); 82% A/18% B (4 min); 75% A/25% B (6 min); 60% A/40% B (6.5–9 min), 5% A/95% B (10–11.5 min), 95% A/5% B (12–13 min). The system operated at a 0.4 mL/min flow rate, a 10 μL injection volume, and a 40°C column oven temperature. The detection wavelength is 326 nm (Table 1).

**TABLE 1:** Standard curve regression equation.

Peaks	Compounds	Standard curve regression equation
1	Caffeic acid	*Y* = 1.359021e5X + 4.216226e3, *R* ^2^ = 0.999987
2	Ferulic acid	*Y* = 1.479405e5X + 1.380338e4, *R* ^2^ = 0.999479
3	Apigenin‐5‐O‐glucoside	*Y* = 3.168104e4X + 3.929982e3, *R* ^2^ = 0.998974
4	Rosmarinic acid	*Y* = 7.913031e4X + 1.607363e4, *R* ^2^ = 0.993956
5	Baicalin	*Y* = 4.145013e4X‐1.870261e4, *R* ^2^ = 0.996856
6	Luteolin	*Y* = 6.791686e4X‐1.675555e4, *R* ^2^ = 0.997261
7	Genistein	*Y* = 2.423445e4X‐1.248783e, *R* ^2^ = 0.994697
8	Apigenin	*Y* = 8.548044e4X‐5.592963e, *R* ^2^ = 0.992417
9	Diosmetin	*Y* = 3.599270e4X, *R* ^2^ = 0.994681

### 2.7 DPPH and ABTS Assay

The methodology of Chen et al. (2025) was implemented with minor modifications. Sample solution (2 mL) with different concentrations (1–200 μg/mL) was mixed with 4 mL of DPPH (1 mmol/L, methanol solution). Subsequently, it was incubated at ambient temperature in the dark for 30 min, and absorbance was measured at 517 nm. (Vc: positive control; methanol: blank). DPPH radical scavenging activity (%) calculated using Formula (1). Calculate the IC50 values using statistical software.

(1) $$\text{Scavenging rate}\;\left(\%\right)=\left(1-\frac{A_{s}-A_{b}}{A_{0}}\right)\times 100$$

where A_s_ was the absorbance of the sample; A_b_ and A_0_ were the absorbance without DPPH and the sample.

The method of Chen et al. (2025) was implemented with minor modifications. Sample solutions (0.1 mL) at concentrations of 1–200 μg/mL were mixed with 0.9 mL of 7 mmol/L ABTS• + solution. The mixture was thoroughly shaken to ensure homogeneity, and then it was placed at ambient temperature in the dark for 10 min, and absorbance was measured at 734 nm (Vc: positive control). ABTS radical scavenging activity (%) calculated using Formula (2):

(2) $$\text{Scavenging rate}\;\left(\%\right)=\left(\frac{A_{c}-A_{t}}{A_{c}}\right)\times 100\%$$

where A_c_ and A_t_ were the absorbance values without and with the sample.

### 2.8 Cell Culture

The RAW264.7 cells were cultured in DMEM supplemented with 10% fetal bovine serum. The RAW264.7 cells were cultured in DMEM supplemented with 10% FBS. Cultures were maintained at 37°C in a humidified incubator with 5% CO_2_. Cells were detached by gentle pipetting after PBS washing, pelleted via centrifugation (1000 rpm, 5 min), resuspended in medium, and then seeded.

### 2.9 CCK‐8 Assay

RAW 264.7 cells were placed in 96‐well plates at 1.5 × 10^5^ cells per well. They were left overnight to attach and grow. Next, some cells were treated with 1 μg/mL LPS for 1 h, while others were not treated (control group). After that, all cells were exposed to different amounts of PSH‐P (5, 10, 20, 50, 100, and 200 μg/mL) and incubated for 24 h. Then, in the dark, 100 μL of CCK‐8 reagent was added to each well. The plates went back into the incubator for 2 h so the reaction could happen. Finally, the absorbance of each well was measured at 450 nm.

### 2.10 Determination of NO, TNF‐α, IL‐1β, and IL‐6

Cells seeded in 6‐well plates were split into Control, LPS, and PSH‐*P* + LPS groups, followed by 24 h incubation. After supernatant removal, each group received specific treatments: the control group was supplemented with 50 μL DMEM; the LPS group received 500 μL DMEM containing 1 μg/mL LPS; while the PSH‐*P* + LPS group underwent pretreatment with graded PSH‐P concentrations (50, 100, 200 μg/mL) for 1 h prior to LPS addition (final concentration 1 μg/mL). All groups were subsequently cultured for an additional 24 h before supernatant collection for quantification of NO, TNF‐α, IL‐1β, and IL‐6. Supernatants were mixed with equal volumes of Griess Reagent I and II and incubated for 10 min at ambient temperature. NO concentration was measured by absorbance at 540 nm.

### 2.11 Determination of ROS Levels

After 8 h of co‐culture in confocal microscopy chambers, the culture medium was aspirated. DCFH‐DA (10 μmol/mL), a fluorescent probe for ROS detection, was added and incubated for 20 min at 37°C. The supernatant was aspirated, and cells were washed three times with serum‐free medium to eliminate extracellular DCFH‐DA. ROS fluorescence was visualized and imaged using a confocal microscope (Leica SP8, Vizsla, Germany).

### 2.12 Determination of mRNA Expression of iNOS and COX‐2

After 8 h of co‐cultivation in 6‐well plates, total RNA was extracted using TriQuick. Complementary DNA (cDNA) was synthesized using a cDNA synthesis kit on the ABI QuantStudio 1 thermal cycler from Thermo Fisher Scientific. With GAPDH serving as an internal reference gene, the relative expression levels of target genes were calculated. Primers are listed below: GAPDH (F: 5′‐TGCCCCCATGTTTGTGATG‐3′; R: 5′‐TGTGGTCATGAGCCCTTCC‐3′); iNOS (F: 5′‐CCGAAGCAAACATCACATTCA‐3′; R: 5′‐GGTCTAAAGGCTCCGGGCT‐3′); COX‐2 (F: 5′‐GCGACATACTCAAGCAGGAGCA‐3′; R: 5′‐AGTGGTAACCGCTCAGGTGTTG‐3′).

### 2.13 Western Blot Assay

After 24 h of co‐cultivation in 6‐well plates, we removed the supernatant and collected the cells. Subsequently, protein extraction and concentration determination were carried out using the BCA Protein Assay Kit. Western blotting was employed to detect the protein expression levels of p65, P‐p65, IκBα, and P‐IκBα, with GAPDH serving as the internal reference. The bands were imaged using a functional imaging system (SCG‐W5000, Saibo Biotechnology, China).

### 2.14 Immunofluorescence

Cells were seeded in laser confocal dishes. The control, LPS, and PSH‐P (200 μg/mL) + LPS groups were incubated for 24 h. Cells were washed twice with PBS, fixed with 3% methanol for 15 min, blocked with immunostaining buffer at ambient temperature for 30 min, and washed three times. They were then incubated with an anti‐p65 primary antibody, washed three times, incubated with a secondary antibody, and stained with DAPI after washing. After another wash, samples were sealed. Slides were imaged using a laser scanning confocal microscope (Leica SP8, Vizsla, Germany).

### 2.15 Statistical Analysis

**FIGURE 1:**
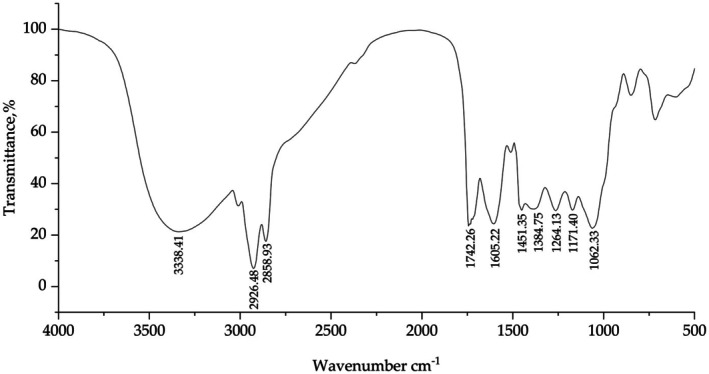
FT‐IR spectrum of PSH‐C.

**FIGURE 2:**
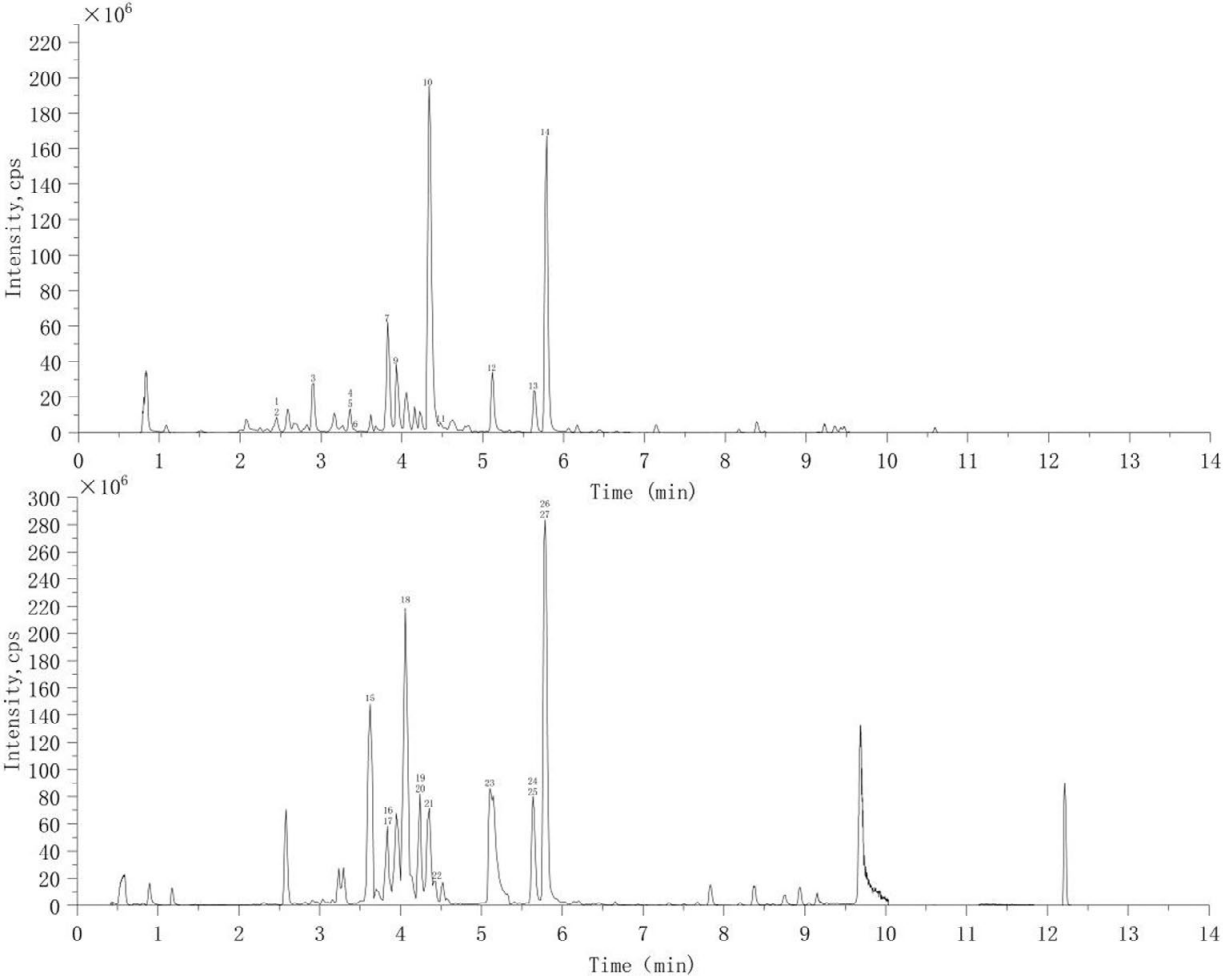
TIC chromatograms of the PSH‐P recorded in negative (top) and positive (bottom) ion modes.

**FIGURE 3:**
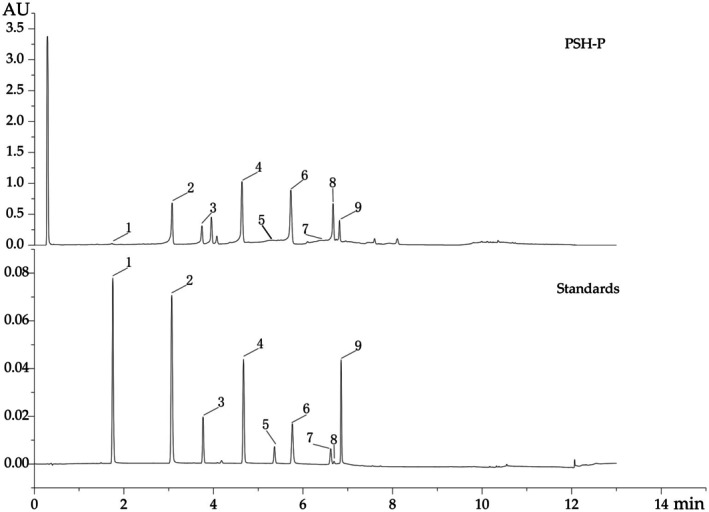
HPLC chromatograms of PSH‐P, and the phenolic standards.

The data was presented as means ± SD obtained from three parallel sets of experiments. The average fluorescence intensity was calculated using ImageJ (1.54f) to quantify the average level of intracellular ROS. The Student's *t*‐test was performed using GraphPad Prism (8.0.2) to analyze the differences between groups. The *p* < 0.05, *p* < 0.01, and *p* < 0.001 indicate significant, highly significant, and extremely significant differences, respectively. Figures 1, 2, 3 were plotted using Origin 2024, while the remaining figures were generated using GraphPad Prism (Tables 2 and 3).

**TABLE 2:** The phenolic compositions of PSH‐P.

Peak	Identification	Rt (min)	Formula	Found at *m/z*	Expected at *m/z*	Error (ppm)	Major fragment ion (*m/z*)
**Phenolic acids**							
1	Gentisic Acid	2.56	C_7_H_6_O_4_	153.01977	153.0193	3.1	109.0292, 108.0211
2	Grevilloside F	2.59	C_15_H_18_O_9_	341.08741	341.0878	1.1	179.0352, 108.0211
3	6‐O‐Caffeoyl‐D‐glucose	2.92	C_15_H_18_O_9_	341.08962	341.0878	5.3	179.0360, 135.0456
4	Caffeic acid	3.37	C_9_H_8_O_4_	179.03598	179.035	5.5	135.0458, 134.0379, 117.0347
5	Vanillic acid	3.39	C_8_H_8_O_4_	167.03599	167.035	5.9	152.0107, 123.0073, 107.0171
6	Syringic acid	3.41	C_9_H_10_O_5_	197.04596	197.0455	2.3	182.0224, 166.9971, 153.0573, 123.0075, 121.0279
9	Ferulic acid	3.94	C_10_H_10_O_4_	193.05101	193.0506	2.1	149.0601, 134.0369, 121.0657
10	Sinapinaldehyde	4.43	C_11_H_12_O_4_	207.06697	207.0663	3.2	192.0425, 177.0197, 149.0233
20	Rosmarinic acid	4.22	C_18_H_16_O_8_	361.08975	361.0918	5.7	163.0378, 135.0423
**Flavonoids**							
7	Kaempferol‐3‐O‐glucuronide	3.83	C_21_H_18_O_12_	461.07269	461.0725	0.4	285.0450
8	Spiraeoside	3.86	C_21_H_20_O_12_	463.08683	463.0882	3	301.0374, 300.0296
12	Luteolin	5.05	C_15_H_10_O_6_	285.04215	285.0405	5.8	151.0046, 133.0306
13	Diosmetin	5.62	C_16_H_12_O_6_	299.05735	299.0561	4.2	284.0336
14	Galangin	5.73	C_15_H_10_O_5_	269.04621	269.0455	2.6	151.0037, 117.0350
15	Scutellarein	3.57	C_15_H_10_O_6_	287.05478	287.055	0.8	153.0178
16	Apigenin‐7‐O‐Gentiobioside	3.74	C_27_H_30_O_15_	595.16175	595.1657	6.6	271.0617
17	Apigenin‐5‐O‐glucoside	3.85	C_21_H_20_O_10_	433.11198	433.1129	2.1	271.0595
19	Kaempferol‐3‐O‐glucoside or Luteolin‐4′‐O‐glucoside	4.2	C_21_H_20_O_11_	449.10587	449.1078	4.3	287.0543
22	Baicalin	4.42	C_21_H_18_O_11_	447.08907	447.0922	7	271.0573
23	Isoscutellarein	5.1	C_15_H_10_O_6_	287.05356	287.055	5	173.0172
24	Genistein	5.6	C_15_H_10_O_5_	271.0592	271.0601	3.3	151.0178, 119.0489
25	Apigenin	5.68	C_15_H_10_O_5_	271.05904	271.0601	3.9	153.0192, 119.0499
26	Scutellarein‐4′‐methyl ether or Rhamnocitrin	5.8	C_16_H_12_O_6_	301.06962	301.0707	3.6	286.0468, 258.0527
**Coumarins**							
11	3,4‐Dihydrocoumarin	4.47	C_9_H_8_O_2_	147.04562	147.0452	2.9	129.0357, 119.0491
18	6‐hydroxycoumarin	4.01	C_9_H_6_O_3_	163.0378	163.039	7.4	135.0452, 117.0349
21	Diosmetin‐7‐O‐glucuronide	4.31	C_22_H_20_O_12_	477.10066	477.1028	4.5	301.0684, 286.0434

**TABLE 3:** The major phenolic compounds and contents in PSH‐P.

Peaks	Compounds	Content (mg/g)
1	Caffeic acid	0.522
2	Ferulic acid	13.778
3	Apigenin‐5‐O‐glucoside	28.922
4	Rosmarinic acid	40.485
5	Baicalin	31.919
6	Luteolin	48.074
7	Genistein	8.308
8	Apigenin	20.632
9	Diosmetin	0.436

## 3 Results

### 3.1 FT‐IR Analysis of PSH‐C

Figure 1 displays the FT‐IR information of PSH‐C. A broad peak observed at 3338.41 cm^−1^ corresponds to the O–H stretching vibration, indicating the presence of abundant hydroxyl‐containing compounds (Bolade et al. 2019). Peaks at 2926.48 cm^−1^ (asymmetric stretching) and 2858.93 cm^−1^ (symmetric stretching) are attributed to aliphatic C–H stretching vibrations, likely originating from the hydrocarbon structures of polyphenol‐derived glucosides (Bolade et al. 2019). The absorption at 1742.26 cm^−1^ is assigned to the C=O stretching vibration of carbonyl groups, suggesting the existence of polyphenolic carbonyl structures (Sinanoglou et al. 2018). Peaks at 1605.22 cm^−1^ and 1451.35 cm^−1^ arise from aromatic C=C skeletal vibrations, while the band at 3010.14 cm^−1^ further confirms aromatic C–H stretching vibrations, collectively supporting the presence of phenolic compounds (Bolade et al. 2019). The signal at 1264.13 cm^−1^ corresponds to C–O stretching vibrations in hydroxylated flavonoids, whereas the peak at 1171.40 cm^−1^ reflects C–O and C–C stretching modes in flavonoids (Sinanoglou et al. 2018). The absorption at 1062.33 cm^−1^ is characteristic of primary alcohol C–O stretching vibrations (Bolade et al. 2019). Additionally, the C–H bending vibration at 1384.75 cm^−1^ may originate from hydrocarbon moieties in polyphenol‐derived glucosides (Andreou et al. 2018). In summary, the results further demonstrate that PSH‐C is rich in diverse classes of polyphenol compounds.

### 3.2 TPC and TFC of PSH‐C and PSH‐P

The contents of TPC and TFC in PSH‐C were 140.92 mg GAE/g extract and 188.09 mg RE/g extract. The contents of TPC and TFC in PSH‐P were 355.26 mg GAE/g E and 501.33 mg RE/g E. Based on TPC measurements, purification with HPD600 macroporous resin resulted in a 2.52‐fold increase in phenolic content purity of PSH‐P compared to PSH‐C. Based on Lee et al. (2013) experiments, high‐purity, high‐content polyphenols and flavonoids could serve as the anti‐inflammatory basis of PSH‐P.

### 3.3 UPLC‐ESI‐MS/MS Analysis of PSH‐P

#### 3.3.1 Phenolic Acids

Peak 1 (Rt: 2.56 min) showed a molecular ion with *m/z* 153.01977 [M−H]^−^ in the mass spectrum. MS^2^ fragmentation yielded characteristic ions with *m/z* 109.0292 [M−H−CO_2_]^−^ and *m/z* 108.0211 [M−H−COOH]^−^. Based on the database entry bearing the formula C_7_H_6_O_4_, the analyte was identified as Gentisic Acid (Udhayaraj et al. 2022). Peak 2 (Rt: 2.59 min) showed a quasi‐molecular ion with *m/z* 341.08741 [M−H]^−^. Characteristic MS^2^ fragments at *m/z* 179.0352 [M−H−C_6_H_10_O_5_]^−^ and *m/z* 108.0211 [M−H−COOH−CO_2_]^−^. The MWDB database matched the formula C_15_H_18_O_9_, identifying the analyte as Grevilloside F. Peak 3 (Rt: 2.92 min) showed a quasi‐molecular ion with *m/z* 341.08962 [M−H]^−^. Characteristic MS^2^ fragments at *m/z* 179.0360 [M−H−C_6_H_10_O_5_]^−^ and *m/z* 135.0456 [M−H−C_6_H_10_O_5_−CO_2_]^−^. Based on the database entry bearing the molecular formula C_15_H_18_O_9_, the analyte was identified as 6‐O‐Caffeoyl‐D‐glucose Acid (Tan et al. 2024). Peak 4 (Rt: 3.37 min) showed a molecular ion with *m/z* 179.03598 [M−H]^−^. MS^2^ fragmentation yielded characteristic ions with *m/z* 135.0458 [M−H−CO_2_]^−^, *m/z* 134.0379 [M−H−COOH]^−^, and *m/z* 117.0347 [M−H−CO_2_−H_2_O]^−^. Based on the database entry bearing the formula C_9_H_8_O_4_, the analyte was identified as Caffeic acid (Goufo et al. 2020; Chen et al. 2017). Peak 5 (Rt: 3.39 min) showed a quasi‐molecular ion with *m/z* 167.03599 [M−H]^−^. Diagnostic MS^2^ fragments included *m/z* 152.0107 [M−H−CH_3_]^−^, *m/z* 123.0073 [M−H−CO_2_]^−^, and *m/z* 107.0171 [M−H−CO_2_−H_2_O]^−^. Based on the database entry bearing the formula C_8_H_8_O_4_, the analyte was identified as Vanillic acid (Goufo et al. 2020). Peak 6 (Rt: 3.41 min) showed a quasi‐molecular ion with *m/z* 197.04596 [M−H]^−^. MS^2^ fragmentation yielded ions with *m/z* 182.0224 [M−H−CH_3_]^−^, *m/z* 166.9971 [M−H−2CH_3_]^−^, *m/z* 153.0573 [M−H−CO_2_]^−^, *m/z* 123.0075 [M−H−CO_2_−2CH_3_]^−^, and *m/z* 121.0279 [M−H−CO_2_−CH_3_OH]^−^. Based on the database entry bearing the formula C_9_H_10_O_5_, the analyte was identified as Syringic acid (Goufo et al. 2020). Peak 9 (Rt: 3.94 min) demonstrated a quasi‐molecular ion with *m/z* 193.05101 [M−H]^−^. Key MS^2^ fragments at *m/z* 149.0601 [M−H−CO_2_]^−^, *m/z* 134.0369 [M−H−CO_2_−CH_3_]^−^, and *m/z* 121.0657 [M−H−C_3_H_4_O_2_]^−^. Based on the database entry bearing the formula C_10_H_10_O_4_, the analyte was identified as Ferulic acid (Huang et al. 2022). Peak 10 (Rt: 4.43 min) showed a quasi‐molecular ion with *m/z* 207.06697 [M−H]^−^. MS^2^ fragmentation yielded characteristic ions with *m/z* 192.0425 [M−H−CH_3_]^−^, *m/z* 177.0197 [M−H−2CH_3_]^−^, and *m/z* 149.0233 [M−H−CO−2CH_3_]^−^. Based on the database entry bearing the formula C_11_H_12_O_4_, the analyte was identified as Sinapinaldehyde. Peak 20 (Rt: 4.22 min) showed a quasi‐molecular ion with *m/z* 361.08975 [M+H]^+^. MS^2^ fragmentation yielded characteristic ions with *m/z* 163.0378 [M+H−C_9_H_10_O_5_]^+^ (cleavage of the ester bond linked to the Danshensu moiety), and *m/z* 135.0423 [M+H−C_9_H_10_O_4_−CO_2_]^+^ (cleavage of the ester bond linked to the caffeic acid moiety, followed by decarboxylation). Based on the database entry bearing the formula C_18_H_16_O_8_, the analyte was identified as Rosmarinic acid (Chen et al. 2017; Razgonova et al. 2022).

#### 3.3.2 Flavonoids

Peak 7 (Rt: 3.83 min) showed a quasi‐molecular ion with m/z 461.07269 [M−H]^−^. Glycosidic bond cleavage generated a fragment at *m/z* 285.0450 [M−H−C_6_H_8_O_6_]^−^. Based on database entry bearing the formula C_21_H_18_O_12_, the analyte was identified as Kaempferol‐3‐O‐glucuronide (Goufo et al. 2020). Peak 8 (Rt: 3.86 min) showed a quasi‐molecular ion with *m/z* 463.08683 [M−H]^−^. MS^2^ fragmentation yielded characteristic ions with m/z 301.0374 [M−H−C_6_H_10_O_5_]^−^ and m/z 300.0296 [M−H−C_6_H_10_O_5_−H]^−^. Based on database entry bearing the formula C_21_H_20_O_12_, the analyte was identified as Spiraeoside (Cai et al. 2023). Peak 12 (Rt: 5.05 min) showed a quasi‐molecular ion with *m/z* 285.04215 [M−H]^−^. Retro‐Diels–Alder (RDA) cleavage produced fragments at *m/z* 151.0046 [M−H−C_8_H_6_O_2_]^−^ and *m/z* 133.0306 [M−H−C_7_H_4_O_4_]^−^. Based on database entry bearing the formula C_15_H_10_O_6_, the analyte was identified as Luteolin (Śliwka‐Kaszyńska et al. 2022). Peak 13 (Rt: 5.62 min) showed a quasi‐molecular ion with *m/z* 299.05735 [M−H]^−^ with a fragment at *m/z* 284.0336 [M−H−CH_3_]^−^. Based on database entry bearing the formula C_16_H_12_O_6_, the analyte was identified as Diosmetin (Di Loreto et al. 2018). Peak 14 (Rt: 5.73 min) demonstrated a quasi‐molecular ion with *m/z* 269.04621 [M−H]^−^. RDA cleavage generated fragments at *m/z* 151.0037 [M−H−C_8_H_6_O]^−^ and *m/z* 117.0350 [M−H−C_7_H_4_O_4_]^−^. Based on database entry bearing the formula C_15_H_10_O_5_, the analyte was identified as Galangin (Zagórska et al. 2024). Peaks 15 and 23 were identified as isomers. Based on database entry bearing the molecular formula C_15_H_10_O_6_, the analyte was identified as Galangin. Peak 15 (Rt: 3.57 min) showed a quasi‐molecular ion with *m/z* 287.05478 [M+H]^+^. RDA cleavage of the C‐ring yielded a fragment at *m/z* 153.0178 [M+H−C_8_H_6_O_2_]^+^. The analyte was identified as Scutellarein (Wei et al. 2024). Peak 23 (Rt: 5.10 min) showed *m/z* 287.05356 [M+H]^+^ with a fragment at *m/z* 153.0172 [M+H−C_8_H_6_O_2_]^+^, assigned as Isoscutellarein (Liu et al. 2018). Peak 16 (Rt: 3.74 min) showed a quasi‐molecular ion with *m/z* 595.16175 [M+H]^+^. Cleavage of the disaccharide moiety (loss of C_12_H_20_O_10_) produced a fragment at *m/z* 271.0617 [M+H−C_12_H_20_O_10_]^+^. Based on database entry bearing the formula C_27_H_30_O_15_, the analyte was identified as Apigenin‐7‐O‐Gentiobioside (Freitas et al. 2019). Peak 17 (Rt: 3.85 min) showed a quasi‐molecular ion with *m/z* 433.11198 [M+H]^+^. MS^2^ fragments were observed at *m/z* 271.0595 [M+H−C_6_H_10_O_5_]^+^. Based on database entry bearing the formula C_21_H_20_O_10_, the analyte was identified as Apigenin‐5‐O‐glucoside (Zhang et al. 2011). Peak 19 (Rt: 4.20 min) showed a quasi‐molecular ion with *m/z* 449.10587 [M+H]^+^, and a MS^2^ fragment at m/z 287.0543 [M+H−C_6_H_10_O_5_]^+^. Based on database entry bearing the formula C_21_H_20_O_11_, the analyte was identified as Kaempferol‐3‐O‐glucoside or Luteolin‐4′‐O‐glucoside (Yang et al. 2014). Peak 22 (Rt: 4.42 min) showed a quasi‐molecular ion with *m/z* 447.08907 [M+H]^+^. Glycosidic bond cleavage produced a fragment at *m/z* 271.0573 [M+H−C_8_H_8_O_6_]^+^. Based on database entry bearing the formula C_21_H_18_O_11_, the analyte was identified as Baicalin (Wang et al. 2018). Peak 24 (Rt: 5.60 min) showed a quasi‐molecular ion with *m/z* 271.0592 [M+H]^+^. RDA cleavage generated fragments at *m/z* 151.0178 [M+H−C_8_H_6_O]^+^ and *m/z* 119.0489 [M+H−C_7_H_4_O_4_]^+^. Based on database entry bearing the formula C_15_H_10_O_5_, the analyte was identified as Genistein (Rashid et al. 2023). Peak 25 (Rt: 5.68 min) showed a molecular ion with *m/z* 271.05904 [M+H]^+^. RDA cleavage generated fragments at *m/z* 153.0192 [M+H−C_8_H_6_O]^+^ and *m/z* 119.0499 [M+H−C_7_H_4_O_4_]^+^. Based on database entry bearing the formula C_15_H_10_O_5_, the analyte was identified as Apigenin (Marta et al. 2023). Peak 26 (Rt: 5.83 min) showed a quasi‐molecular ion with *m/z* 301.06938 [M+H]^+^, and MS^2^ fragments at *m/z* 286.0468 [M+H−CH_3_]^+^ and *m/z* 258.0527 [M+H−CO−CH_3_]^+^. Based on database entry bearing the formula C_16_H_12_O_6_, the analyte was identified as Scutellarein‐4′‐methyl ether or Rhamnocitrin (Chernonosov et al. 2017).

#### 3.3.3 Coumarins

Peak 11 (Rt: 4.47 min) showed a molecular ion with *m/z* 147.04562 [M−H]^−^. MS^2^ fragmentation generated fragments at *m/z* 129.0357 [M−H−H_2_O]^−^ and *m/z* 119.0491 [M−H−CO]^−^. Based on database entry bearing the formula C_9_H_8_O_2_, the analyte was identified as 3,4‐Dihydrocoumarin (Zheng et al. 2017). Peak 18 (Rt: 4.01 min) showed a quasi‐molecular ion with *m/z* 163.0378 [M+H]^+^. MS^2^ fragments included *m/z* 135.0452 [M+H−CO]^+^, *m/z* 117.0349 [M+H−CO−H_2_O]^+^, and *m/z* 117.0349 [M+H−2CO−H_2_O]^+^. Based on database entry bearing the formula C_9_H_6_O_3_, the analyte was identified as 6‐hydroxycoumarin (Li et al. 2023). Peak 21 (Rt: 4.31 min) demonstrated a molecular ion with *m/z* 477.10066 [M+H]^+^. Cleavage of the glucuronide moiety (loss of C_6_H_8_O_6_) produced fragments at *m/z* 301.0684 [M+H−C_6_H_8_O_6_]^+^ and *m/z* 286.0434 [M+H−C_6_H_8_O_6_−CH_3_]^+^. Based on database entry bearing the formula C_22_H_20_O_12_, the analyte was identified as Diosmetin‐7‐O‐glucuronide (Xu et al. 2024).

### 3.4 UPLC Analysis of PSH‐P

Chromatographic profiling (Figure 3) revealed PSH‐P to be predominantly composed of phenolic acids and flavonoids. Quantitative analysis identified luteolin (48.074 mg/g) and rosmarinic acid (40.485 mg/g) as the major constituents, followed by baicalin and apigenin‐5‐O‐glucoside. These high‐content, major polyphenol components will be the primary contributors to the high anti‐inflammatory activity demonstrated by PSH‐P. Notably, apigenin‐5‐O‐glucoside was first characterized in *Perilla frutescens* (L.) Britt.

### 3.5 DPPH and ABTS Radical Scavenging Capacities

**FIGURE 4:**
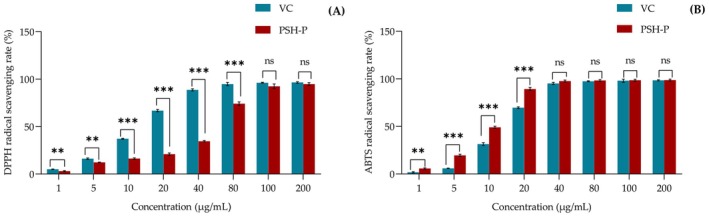
(A) DPPH radical scavenging activity; (B) ABTS radical scavenging activity. Data are presented as mean ± SD (*n* = 3). ns, *p* > 0.05, **p < 0.01, ***p < 0.001.

In Figure 4A, PSH‐P (80 μg/mL) had > 90% scavenging, IC_50_ = 36.97 μg/mL, weaker than Vc (IC_50_ = 13.78 μg/mL). Notably, PSH‐P displayed significantly enhanced ABTS radical neutralization efficacy compared to Vc. As shown in Figure 4B, PSH‐P (20 μg/mL) showed superior ABTS radical scavenging (90% inhibition), with an IC_50_ of 8.82 μg/mL, versus 13.99 μg/mL for Vc. The pronounced ABTS and DPPH radical scavenging capacities exhibited by PSH‐P are likely attributable to its abundant polyphenolic constituents.

### 3.6 Effect of PSH‐P on RAW 264.7 Cell Viability

**FIGURE 5:**
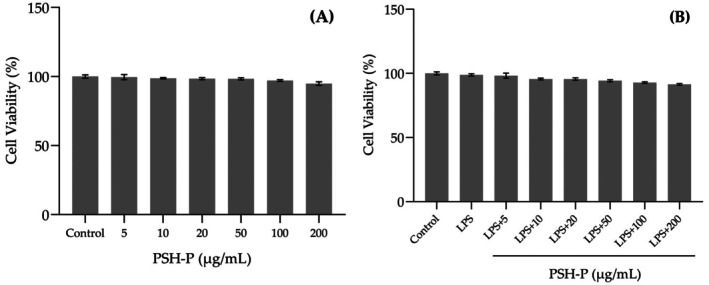
Effect of PSH‐P on RAW264.7 cell viability. (A) and (B) show the cytotoxic effects of PSH‐P on RAW264.7 cells without and with LPS (1 μg/mL). Data are presented as mean SD (*n* = 3).

The results of the Cell Cytotoxicity Assay depicted in Figure 5 indicate that the viability of RAW264.7 cells (both LPS‐pretreated and non‐pretreated) exceeded 90% after the addition of PSH‐P at concentrations ranging from 5 to 200 μg/mL. This demonstrates that PSH‐P is noncytotoxic. Therefore, subsequent experiments utilized PSH‐P at 50, 100, and 200 μg/mL to evaluate its anti‐inflammatory activity.

### 3.7 In Vitro Anti‐Inflammatory Activity

**FIGURE 6:**
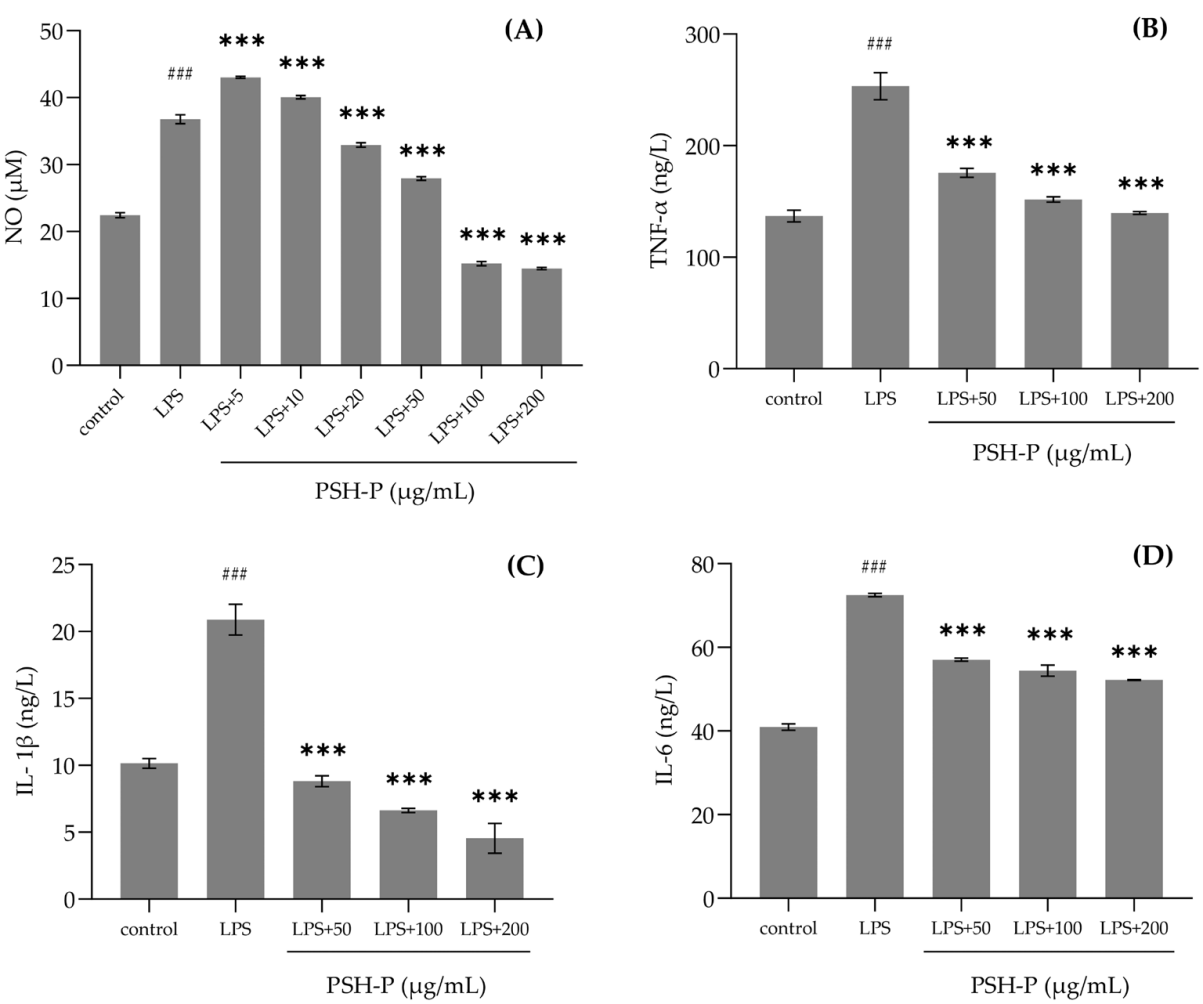
Effects of PSH‐P on LPS‐induced secretion of NO (A), TNF‐α (B), IL‐1β (C), and IL‐6 (D) in RAW 264.7 cells. Data are presented as mean ± SD (*n* = 3). ^###^ *p* < 0.001 versus control group; ***p < 0.001 versus LPS treatment group.

Numerous studies have demonstrated that NO release, as an inflammatory mediator, can effectively serve as an indicator of inflammatory conditions. The experimental findings from Griess experiments show that LPS‐induced RAW264.7 cells exhibited a significant 63.8% elevation in NO production, which was suppressed by PSH‐P treatment through concentration‐dependent mechanisms, and PSH‐P at 100 and 200 μg/mL exhibits stronger inhibitory effects (Figure 6A).

The study quantitatively measured the release of TNF‐α, IL‐1β, and IL‐6 from RAW264.7 cells. ELISA results demonstrated baseline cytokine levels of 136.88 ng/L (TNF‐α), 10.15 ng/L (IL‐1β), and 40.93 ng/L (IL‐6) in control groups, while LPS‐stimulated groups exhibited significantly elevated concentrations of 253.34 ng/mL, 20.89 ng/L, and 72.50 ng/L, respectively. PSH‐P (50, 100, 200 μg/mL) treatment induced concentration‐dependent suppression of these pro‐inflammatory mediators, achieving statistically significant reductions compared to the LPS group (*p* < 0.001). These results unequivocally confirm that PSH‐P can alleviate the excessive secretion of inflammatory cytokines in RAW264.7 cells (Figure 6B–D).

**FIGURE 7:**
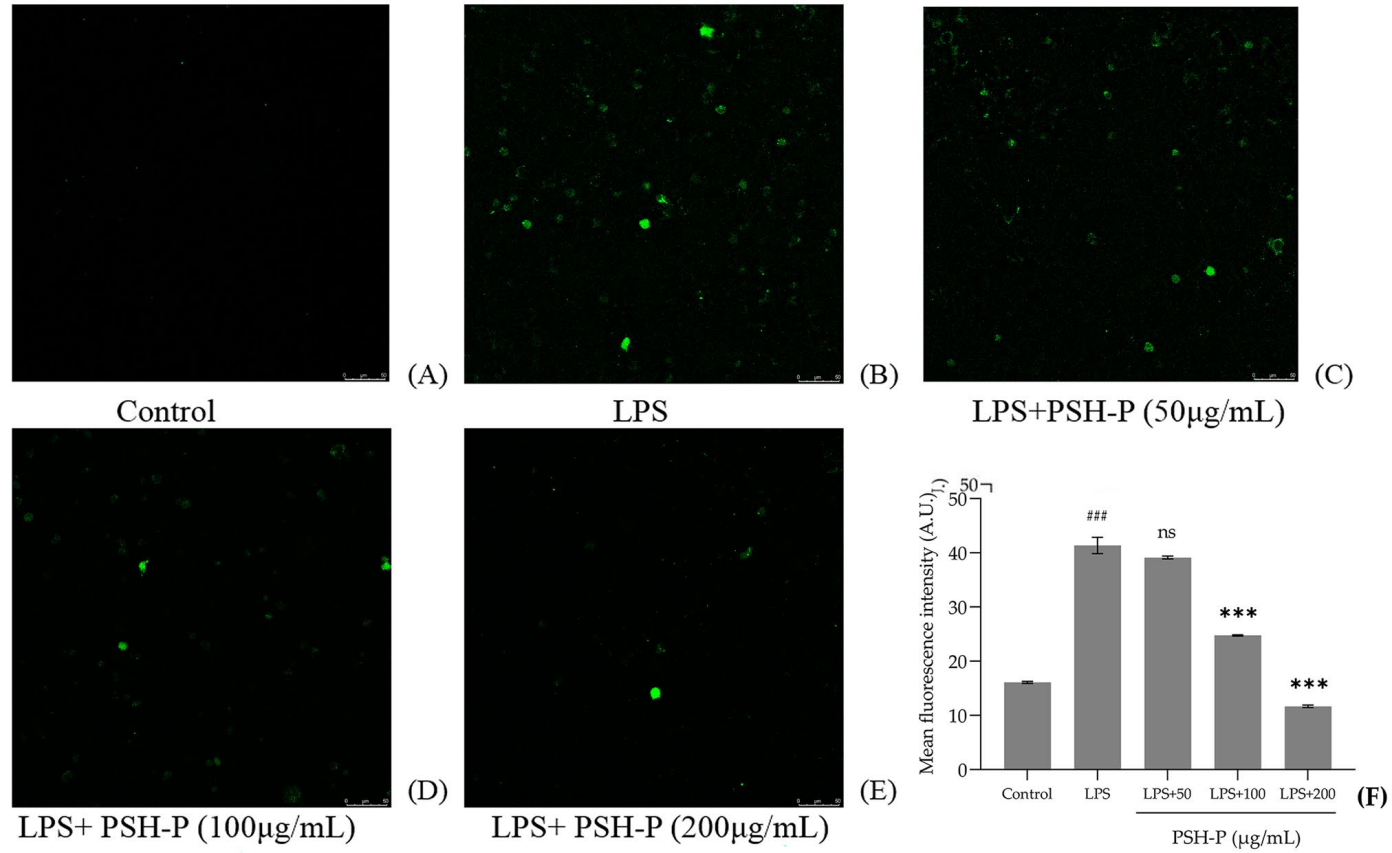
Detection of intracellular ROS levels in RAW264.7 cells. (A–E) Fluorescence intensity of each group. (F) Comparison of ROS levels among groups. Data are presented as mean ± SD (*n* = 3). ^###^ *p* < 0.001 versus control group; ***p < 0.001 versus LPS treatment group.

As shown in Figure 7, the LPS group exhibited a highly significant elevation in ROS‐associated fluorescence intensity relative to the control (*p* < 0.001). Compared with the LPS group, PSH‐P treatment induced a concentration‐dependent decrease in fluorescence intensity of intracellular ROS levels. Furthermore, 100 and 200 μg/mL PSH‐P significantly suppressed ROS generation (*p* < 0.001).

**FIGURE 8:**
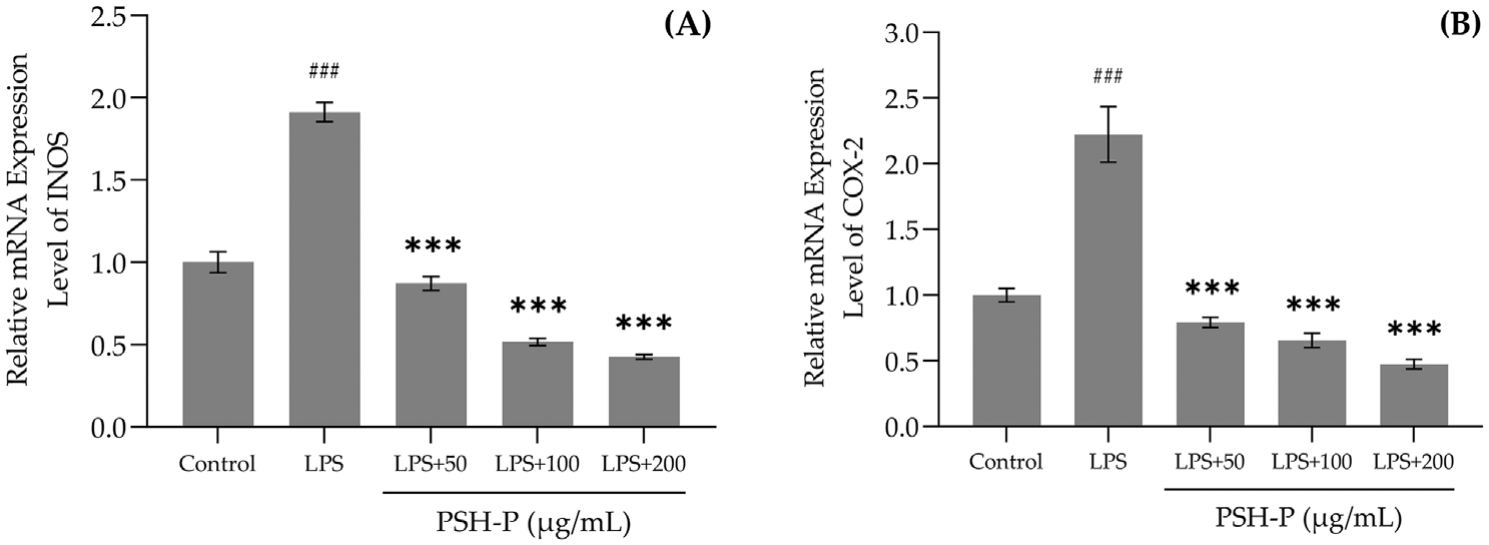
(A) iNOS and (B) COX‐2 mRNA levels. Data are presented as mean SD (*n* = 3). ^###^ *p* < 0.001 versus control group; ***p < 0.001 versus LPS treatment group.

Figure 8 shows that LPS stimulation markedly increased the expression of iNOS and COX‐2, whereas PSH‐P treatment markedly downregulated their expression. This demonstrates that PSH‐P can significantly inhibit LPS‐induced oxidative inflammation.

### 3.8 Effect of PSH‐P on the NF—κB Signaling Pathway

**FIGURE 9:**
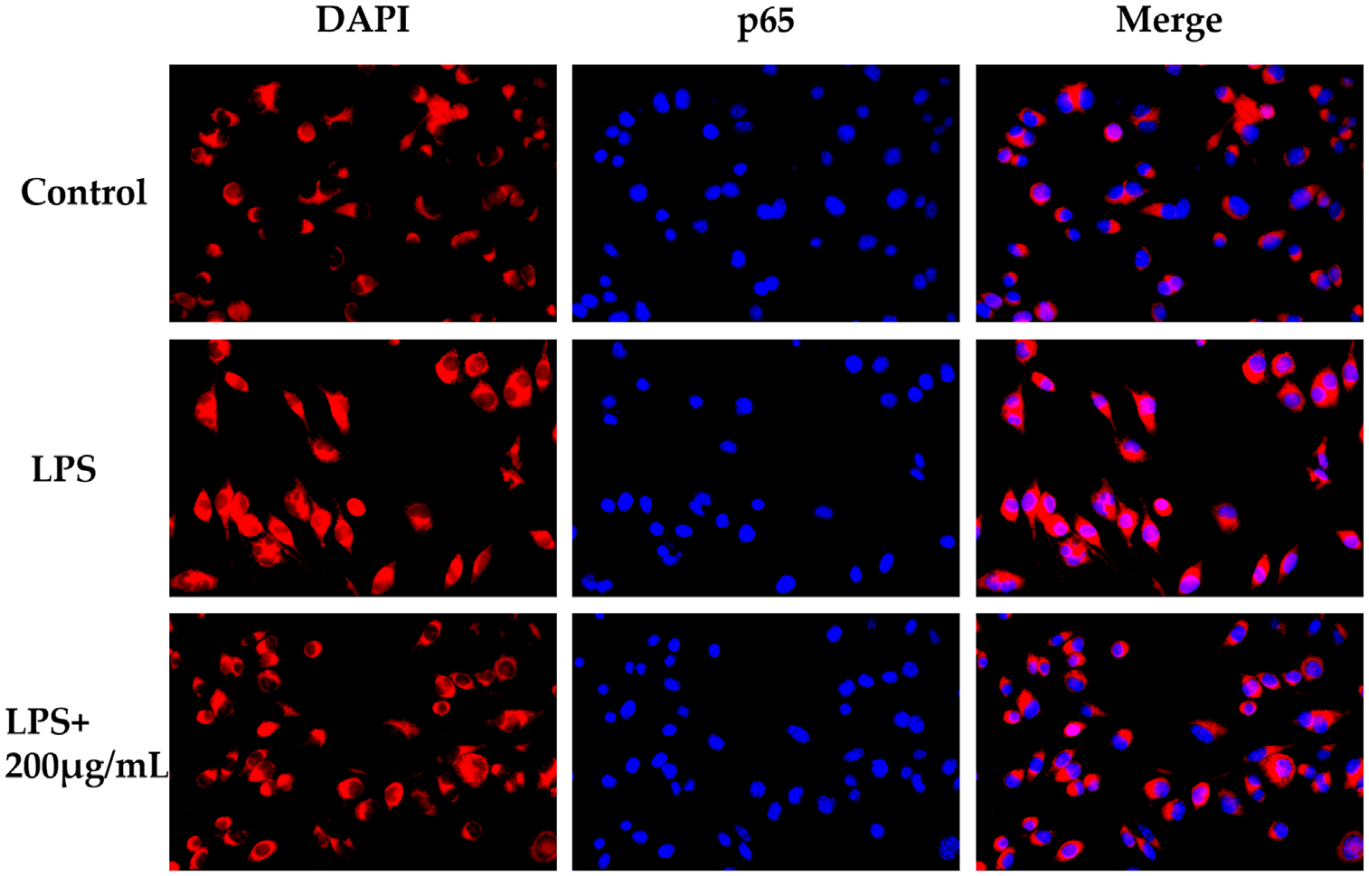
Effect of PSH‐P on LPS‐induced p65 nuclear translocation. Blue = DAPI (nuclei), Red = p65.

Figure 9 reveals distinct subcellular p65 distributions: control cells showed exclusively cytoplasmic red fluorescence (p65) without nuclear penetration in DAPI‐stained nuclei, whereas LPS‐induced cells displayed intense nuclear fluorescence, indicating p65 translocation. This LPS‐induced nuclear translocation was significantly inhibited by 200 μg/mL PSH‐P.

**FIGURE 10:**
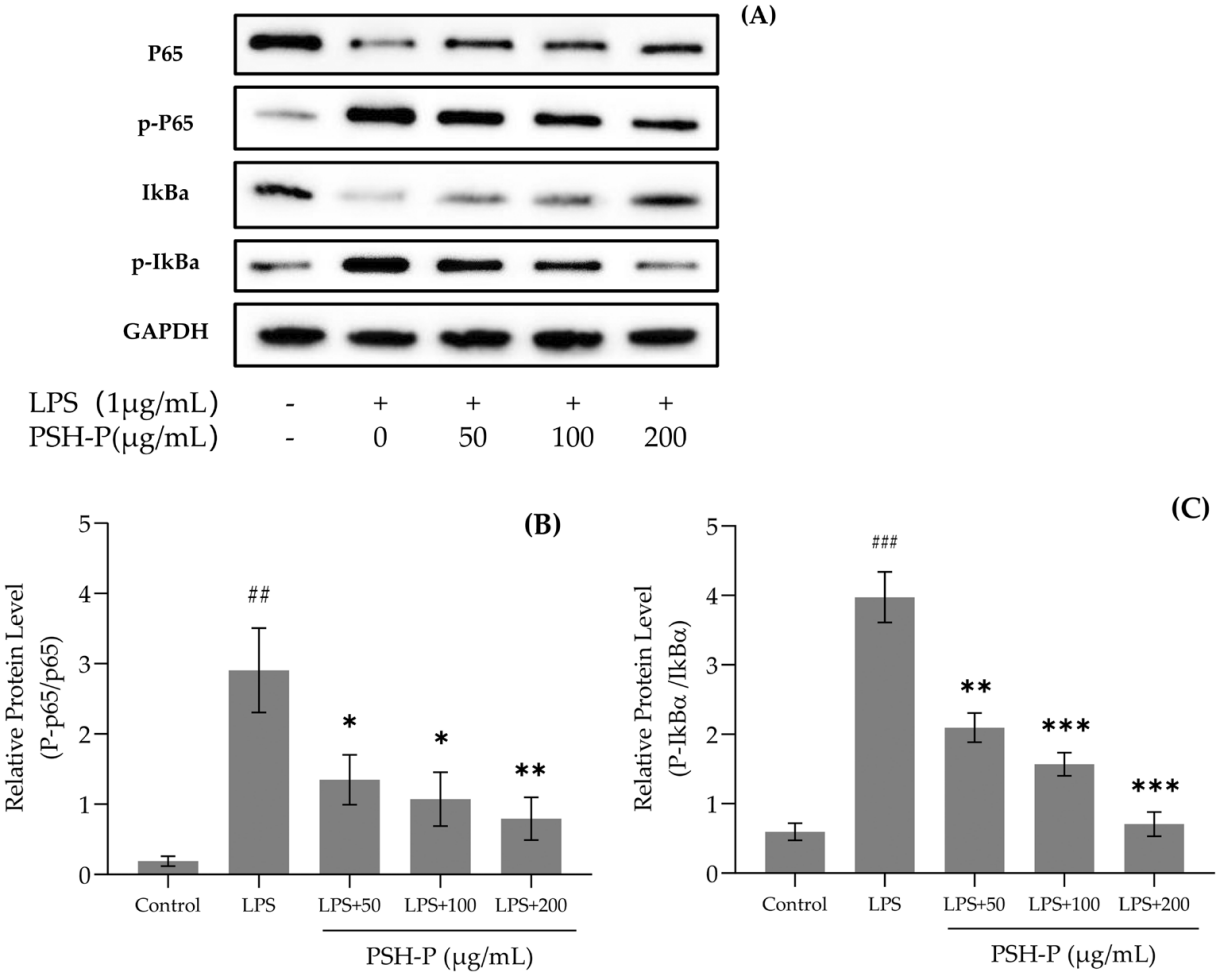
Effect of PSH‐P on LPS‐induced phosphorylation of NF‐κB pathway proteins in RAW264.7 cells. (A) Western blot for p65, IκBα, and their phosphorylation; (B, C) Relative phosphorylation levels of p65 and IκBα. Data are presented as mean ± SD (*n* = 3). ^##^ *p* < 0.01, ^###^ *p* < 0.001 versus control group; *p < 0.05, **p < 0.01, ***p < 0.001 versus LPS treatment group.

Western blot analysis of cytoplasmic p65 and IκBα revealed LPS stimulation significantly enhanced phosphorylation (P‐p65 and P‐IκBα) compared to controls (Figure 10). PSH‐P dose‐dependently inhibited this phosphorylation, with 200 μg/mL treatment reducing P‐p65/p65 and P‐IκBα/IκBα ratios by 72.70% and 82.26%, respectively.

## 4 Discussion

Ultrasound‐assisted organic solvent extraction has been confirmed as an effective method for polyphenol extraction. In their study on *Sophora japonica* , Younis et al. (2025) found that conventional ethanol extraction alone achieved a TPC of 51.18 mg GAE/g E, whereas ultrasound‐assisted extraction significantly enhanced the TPC to 65.57 mg GAE/g E. We employed an ultrasound‐assisted extraction method, utilizing ethanol and water as solvents to extract polyphenolic compounds from PSH. FT‐IR spectroscopy effectively identifies chemical bonds and functional groups in substances, thereby enabling the determination of characteristic peaks specific to polyphenolic compounds. We further confirmed the presence of polyphenolic compounds in PSH‐C through FT‐IR. Macroporous resins are among the most effective and commonly used methods for the purification of plant polyphenols (Gao et al. 2018). We purified PSH‐C using HPD600 macroporous resin through dynamic adsorption–desorption steps to obtain PSH‐P. The study revealed that the purity of PSH‐P was increased by 2.52‐fold compared to the PSH‐C. Coincidentally, Zhang et al. (2021) also utilized HPD600 to separate and purify polyphenolic substances in the *Pinus koraiensis* pinecone shell‐coated film, and established the optimal purification conditions.

Using UPLC‐ESI‐MS/MS coupled with database matching and literature validation, we identified 26 polyphenolic compounds in PSH‐P, including 9 phenolic acids, 14 flavonoids, and 3 coumarins. Notably, the compositional profile exhibited significant overlap with previously reported polyphenolic constituents in *Perilla frutescens* derivatives, corroborating the reproducibility of phytochemical characterization across studies (Hooshmand et al. 2015). The polyphenolic compounds in PSH‐P predominantly exist as parent polyphenols and their derivatives, with the latter primarily in the form of glycosidic conjugates. The result is in contrast to the findings of Razgonova et al. (2022) research. Due to the presence of isomers in natural products, which share identical molecular weights and exhibit similar molecular structures, retention times, and fragmentation patterns, it remains challenging to differentiate them using mass spectrometry alone. A representative example includes the structural isomers Kaempferol‐3‐O‐glucoside and Luteolin‐4′‐O‐glucoside. For a more accurate identification of polyphenolic compounds in PSH‐P, we employed HPLC with reference standards to quantify nine major polyphenols (including three phenolic acids and six flavonoids). Similar to *Perilla frutescens* , PSH‐P was also abundant in rosmarinic acid, baicalin, luteolin, and apigenin, square with the findings of Qi‐ping et al. (2021) and Lee et al. (2013). Notably, we have identified apigenin‐5‐O‐glucoside from *Perilla frutescens* for the first time, a discovery that constitutes a novel contribution to the phytochemical characterization of this species.

DPPH and ABTS assays are critical for evaluating antioxidant activity in natural products and screening potential antioxidants (Chumphukam et al. 2018). Scavenging free radicals can block the inflammatory cascade. The results demonstrated that PSH‐P exhibits potent antioxidant activity, particularly in ABTS radical scavenging, which outperformed Vc under the tested conditions. These findings suggest that the observed DPPH and ABTS radical scavenging effects of PSH‐P may derive from the hydrogen/electron donation ability of its phenolic compounds. Based on the aforementioned findings, PSH‐P demonstrates significant potential for application as an effective antioxidant in both the food industry and therapeutic/pharmaceutical sectors.

This study revealed that PSH‐P exhibited no cytotoxicity toward RAW 264.7 cells while simultaneously inhibiting the release of nitric oxide (NO) and the production of pro‐inflammatory cytokines (e.g., TNF‐α, IL‐1β, and IL‐6). Collectively, these results demonstrate the potent anti‐inflammatory effects of PSH‐P, aligning with previous reports on the anti‐inflammatory properties of jujube peel polyphenols (Shen et al. 2022). ROS are oxygen‐containing chemical reactive molecules, which are considered a factor in the inflammatory responses induced by LPS (Dong and Yuan 2018). ROS trigger NF‐κB pathway activation via IκBα degradation, subsequently inducing the transcription of pro‐inflammatory genes. This process establishes a self‐amplifying inflammation‐oxidative stress circuit. This study demonstrated that high concentrations of PSH‐C effectively suppressed intracellular ROS generation, thereby demonstrating their anti‐inflammatory capabilities. The expression of iNOS and the production of COX serve as crucial indicators of the oxidative inflammatory state in cells. iNOS determines NO production; as an inducible subtype of COX, COX‐2 plays a pivotal role in prostaglandin biosynthesis by mediating the transformation of arachidonic acid. Both are critically involved in inflammatory responses. Therefore, inflammation may be mitigated by inhibiting the expression of iNOS and COX‐2 (Xu et al. 2022). This study demonstrates that PSH‐P treatment potently suppresses iNOS expression and NO production while concurrently inhibiting COX‐2 expression, thereby exerting anti‐inflammatory capabilities. In normal cells, NF‐κB complexes maintain cytoplasmic sequestration via IκBα binding. Upon LPS stimulation, IκB kinase (IKK) activation catalyzes IκBα phosphorylation, priming it for ubiquitin‐dependent proteasomal degradation. This facilitates the nuclear import of the canonical p50‐p65 NF‐κB dimer, provoking transcriptional upregulation of inflammatory mediators including iNOS and COX‐2 (Dai et al. 2018). NF‐κB activation transcriptionally upregulates pro‐inflammatory factors (Ma et al. 2021). Our findings demonstrate that PSH‐P effectively suppresses LPS‐induced phosphorylation of p65 and IκBα proteins in the NF‐κB pathway and blocks the nuclear translocation of p65. This mechanistically explains its anti‐inflammatory action at the cellular level. Our results systematically expound the nutraceutical properties of PSH and advocate its utilization as a sustainable resource for natural antioxidants and anti‐inflammatory compounds in functional foods and pharmaceuticals.

## Author Contributions

**Di Xie:** conceptualization (equal), formal analysis (equal), funding acquisition (equal), methodology (equal), project administration (equal), writing – original draft (equal). **Bingcong Wang:** data curation (equal), formal analysis (equal), software (equal). **Yinlu Gao:** supervision (equal), validation (equal), visualization (equal), writing – review and editing (equal). **Yanrong Zhang:** resources (equal), supervision (equal), validation (equal), writing – review and editing (equal).

## Supporting information


**Appendix S1:** fsn370847‐sup‐0001‐AppendixS1.docx.

## Data Availability

The authors confirm that the data supporting the findings of this study are available within the article.
