# Supplementary material for: Perilla Seed Hulls Polyphenol Extract: Molecular Characterization and Suppression of LPS‐Induced Inflammation in RAW 264.7 Cells via NF‐κB Signaling Pathway
Source: Food Sci Nutr. 2025 Sep 3;13(9):e70847. doi: 10.1002/fsn3.70847 (PMC12406173; doi:10.1002/fsn3.70847)
Supplement: Supplementary file 1 — Appendix S1: fsn370847‐sup‐0001‐AppendixS1.docx. [file FSN3-13-e70847-s001.docx]

**Supporting Information**

**Perilla Seed Hull Polyphenol Extract: Molecular Characterization and Suppression of LPS-Induced Inflammation in RAW 264.7 Cells via NF-κB Signaling Pathway**

**S1 Perilla Seed Hull Polyphenol Extraction and Purification**

**S1.1 Extraction of Polyphenols**

**S1.1.1 Single-Factor Experiments**

Accurately weigh 10.0 g of defatted perilla seed hull powder into a conical flask. Add an ethanol-water solution and perform ultrasonic-assisted extraction for a specified duration. After allowing the mixture to stand, collect the supernatant and centrifuge at 4000 r/min for 10 min. Determine the polyphenol extraction yield from perilla seed hulls using the Folin-Ciocalteu colorimetric method.

The effects of ethanol concentration, ultrasonic power, extraction time, and liquid-to-solid ratio on polyphenol extraction yield were investigated using initial fixed parameters of 60% ethanol (v/v), 400 W ultrasonic power, 30 min extraction time, and 30:1 mL/g liquid-to-solid ratio, followed by one-factor-at-a-time experiments varying individual parameters across designated ranges: ethanol concentration (40-80% v/v), ultrasonic power (200-600 W), extraction time (10-50 min), and liquid-to-solid ratio (30:1 to 70:1 mL/g).

**S1.1.2 Response Surface Methodology Optimization Design**

Based on the single-factor experiments, the response surface methodology (RSM) was employed using a Box-Behnken central composite design through Design-Expert software (Version 8.0.6). Ethanol concentration (A), ultrasonic power (B), extraction time (C), and liquid-to-solid ratio (D) were selected as the four independent factors. Each factor was tested at three levels, with polyphenol content of perilla seed hulls serving as the response variable. The experimental design matrix is presented in Table S1.

Table S1. Three-factor three-level response surface center combination experiment design

| Level | Factor | | | |
| --- | --- | --- | --- | --- |
|  | Ethanol concentration (A)/(%) | Ultrasonic power (B)/(W) | Extraction time (C)/(min) | Liquid-to-solid ratio (D)/（v/w） |
| -1 | 50 | 300 | 20 | 30 |
| 0 | 60 | 400 | 30 | 40 |
| 1 | 70 | 500 | 40 | 50 |

**S1.2 Purification of polyphenols from PSH-C**

**S1.2.1.Adsorbents Preparation**

The purification experiments were conducted using six macroporous resins: D101, HPD100, AB-8, HPD400, HPD600, and NKA-Ⅱ. Prior to use, the resins were fully swollen by soaking in anhydrous ethanol for 24 hours, rinsed with deionized water until no ethanol odor remained and the effluent became clear, then treated with 5% hydrochloric acid for 6 hours, rinsed with distilled water to neutrality, followed by soaking in 5% sodium hydroxide solution for 6 hours, and finally rinsed again with distilled water to neutrality.

**S1.2.2Screening of Suitable Adsorbents**

The experimental procedure was carried out according to the method described by Jin G et al., with appropriate modifications(Jin G et al.2022).

Determine the mass of each of the six pre-treated macroporous resin samples (1 g each) and add 20 mL of PSH-C solution (1 mg/mL) to a 250 mL conical flask. Place the flasks in a constant-temperature shaking incubator and allow adsorption to proceed at 25 °C with a shaking speed of 120 rpm for 24 hours. After adsorption is complete, filter the resin and measure the polyphenol concentration in the supernatant. Once the resin is saturated, wash it with 150 mL of deionized water, filter, and transfer it to a clean 250 mL conical flask. Add 20 mL of 70% ethanol solution and subject the mixture to desorption at 25 °C with a shaking speed of 120 rpm for 24 hours. Finally, collect 1 mL of the eluate and measure its polyphenol concentration. The adsorption and desorption rates are calculated as follows:

$Absorption rate(\%)=\frac{C_{0}-C_{1}}{C_{0}}\times100\%$ (1)

$Desorption rate(\%)=\frac{C_{2}\times V_{2}}{\left( C_{0}-C_{1} \right)\times V_{0}}\times100\%$ (2)

where C_0_ is the concentration of polyphenols before adsorption, C_1_ is the concentration of polyphenols after adsorption, C_2_ is the concentration of polyphenols after desorption, V_0_ is the volume of adsorption solution, V_2_ is the volume of desorption solution.

**S1.2.3 Dynamic absorption and desorption**

The experimental procedure was carried out according to the method described by Gao et al.(Gao Net al.2018), with appropriate modifications. Dynamic adsorption and desorption experiments were conducted at room temperature using pre-treated HPD 600 macroporous resin packed into a chromatography column (16 × 300 mm) via wet packing (bed volume, BV = 40 mL). PSH-C solutions at varying concentrations (1, 2, 3, 4, 5 mg/mL) were passed through the column at flow rates of 0.1, 0.5, 1, 1.5, and 2 mL/min. The process was terminated when the total phenolic content (TPC) in the effluent reached 10% of the initial concentration, marking the breakthrough point. The resin was then rinsed with deionized water at 1.5 mL/min until the effluent became colorless. Desorption was performed using ethanol solutions (40%, 50%, 60%, 70% v/v) at flow rates of 0.25, 0.5, 1, 1.5, and 2 mL/min. The effects of sample concentration, sample flow rate, eluent ethanol concentration, and elution flow rate on polyphenol adsorption and desorption were systematically evaluated.

**S2 Results**

**S2.1 Extraction of Polyphenols**

**S2.1.1 Analysis of Single-Factor Experimental Results**

**S2.1.1.1 Effect of Ethanol Concentration**

As shown in Figure S1(A), the extraction yield of polyphenols from perilla seed hulls progressively increased with rising ethanol volume fraction, reaching a maximum of 26.429 mg/g at 60% (v/v), beyond which the yield gradually declined. This trend may be attributed to higher proportions of aqueous phase at lower ethanol concentrations (<60%) solubilizing water-soluble impurities that competitively inhibited polyphenol dissolution, while at ≥70% ethanol, protein denaturation impeded bound phenolic release and elevated lipophilic impurities acted as competitive inhibitors. Consequently, 60% ethanol volume fraction was selected as optimal due to observed polyphenol dissolution saturation at this concentration.

**S2.1.1.2Effect of Ultrasonic Power**

As depicted in Figure S1(B), the extraction yield of perilla seed hull polyphenols progressively increased with higher ultrasonic power, reaching a maximum value of 26.767 mg/g at 400 W. Beyond this power level, the yield gradually decreased, likely due to excessive ultrasonic energy generating solution temperatures that compromised polyphenol stability through thermal degradation and molecular structural disruption. Consequently, 400 W was selected as the optimal ultrasonic power parameter.

**S2.1.1.3 Effect of Extraction Time**

As illustrated in Figure S1(C), the extraction yield of perilla seed hull polyphenols increased progressively with prolonged extraction time, reaching a peak value of 26.822 mg/g at 30 min. Within the initial 30-minute period, enhanced cellular disruption improved polyphenol release efficiency, after which the yield plateaued. This stabilization likely indicates near-complete extraction of polyphenols, while further ultrasonic treatment elevated solution temperatures causing structural degradation of polyphenols. Consequently, 30 min was selected as the optimal extraction duration.
**S2.1.1.4 Effect of Extraction Time**

As demonstrated in Figure S1(D), the extraction yield of perilla seed hull polyphenols increased progressively within the liquid-to-solid ratio range of 30:1 to 40:1 (mL/g), reaching a maximum value of 27.224 mg/g at 40:1 (mL/g). Beyond this ratio, the yield gradually decreased, likely attributable to saturation of extractable polyphenols at the 40:1 ratio where further solvent increases promoted dissolution of competing impurities while providing no extraction benefit. This phenomenon not only reduces polyphenol recovery efficiency but also constitutes unnecessary solvent consumption. Consequently, the 40:1 (mL/g) liquid-to-solid ratio was selected as optimal.


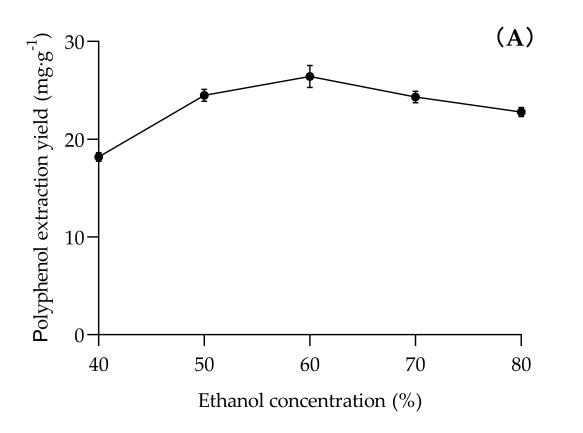

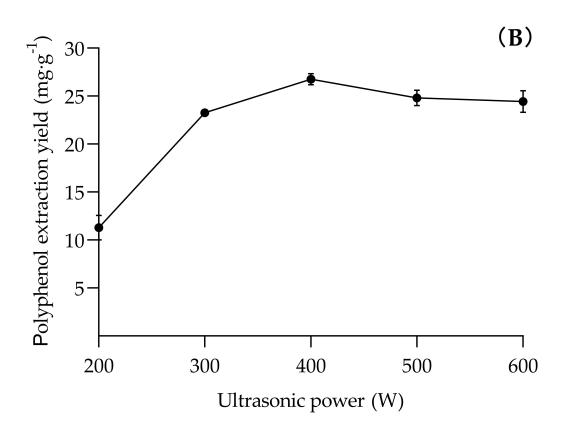


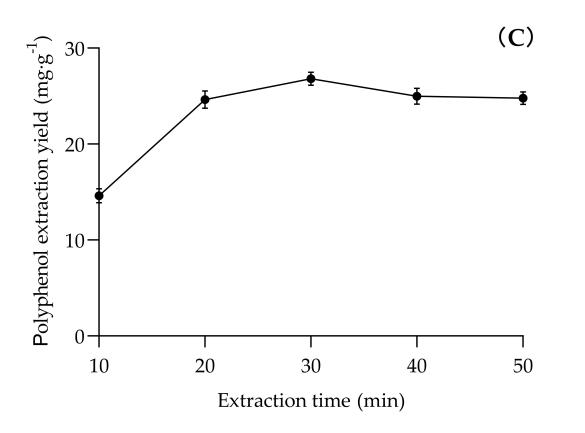

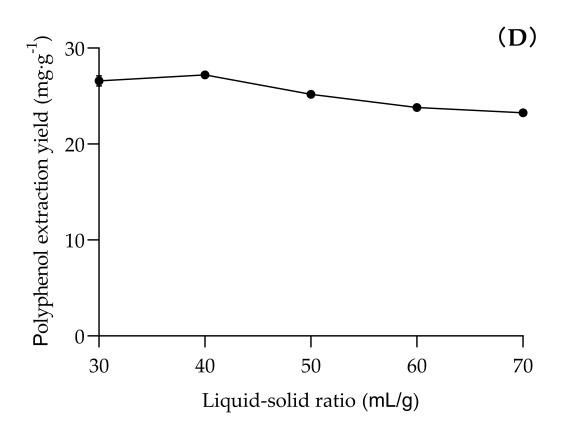


Figure S1. Effects of ultrasonic extraction parameters on the polyphenol extraction rate. (A) Ethanol concentration, (B) Ultrasonic power, (C) Extraction time, (D) Liquid-to-solid ratio. Error bars represent standard deviation (n = 3).

**S2.1.2 Analysis of Response Surface Optimization Results**

A Box-Behnken design was employed to optimize the extraction of phenolic compounds from PSH using ultrasound-assisted extraction technology, focusing on four variables: ethanol concentration (X1), ultrasonic power (X2), extraction time (X3), and liquid-solid ratio (X4). The experimental values of TPC obtained for each run are summarized in Table S2. The results demonstrated that ultrasonic treatment significantly enhanced the release of polyphenols into the solvent, with TPC ranging from 19.24 to 27.97 (mg GAE/g E).

Table S2. Box–Behnken design with natural and coded values (parenthesis) of the conditions of extraction and the experimental results obtained for TPC expressed with the average and the standard deviation.

| Run | Independent Factors | | | | Response  TPC (mg GAE/g E ) |
| --- | --- | --- | --- | --- | --- |
|  | X_1_ | X_2_ | X_3_ | X_4_ |  |
| 1 | 50(-1) | 300(-1) | 30(0) | 40(0) | 20.37±0.4 |
| 2 | 70(1) | 500(1) | 30(0) | 40(0) | 19.52±1.07 |
| 3 | 50(-1) | 500(1) | 30(0) | 40(0) | 20.56±0.76 |
| 4 | 60(0) | 500(1) | 30(0) | 40(0) | 23.58±0.34 |
| 5 | 60(0) | 400(0) | 20(-1) | 30(-1) | 25.29±0.95 |
| 6 | 60(0) | 400(0) | 40(1) | 30(-1) | 24.68±0.5 |
| 7 | 60(0) | 400(0) | 20(-1) | 50(1) | 25.43±0.31 |
| 8 | 60(0) | 400(0) | 40(1) | 50(1) | 23.36±0.89 |
| 9 | 50(-1) | 400(0) | 30(0) | 30(-1) | 25.18±1.18 |
| 10 | 70(1) | 400(0) | 30(0) | 30(-1) | 24.43±0.56 |
| 11 | 50(-1) | 400(0) | 30(0) | 50(1) | 23.63±1.12 |
| 12 | 70(1) | 400(0) | 30(0) | 50(1) | 25.33±0.44 |
| 13 | 60(0) | 300(-1) | 20(-1) | 40(0) | 20.27±0.88 |
| 14 | 60(0) | 500(1) | 20(-1) | 40(0) | 21.94±1.21 |
| 15 | 60(0) | 300(-1) | 40(1) | 40(0) | 20.85±1.12 |
| 16 | 60(0) | 500(1) | 40(1) | 40(0) | 21±0.65 |
| 17 | 50(-1) | 400(0) | 20(-1) | 40(0) | 24.14±0.55 |
| 18 | 70(1) | 400(0) | 20(-1) | 40(0) | 21.44±0.92 |
| 19 | 50(-1) | 400(0) | 40(1) | 40(0) | 19.24±0.98 |
| 20 | 70(1) | 400(0) | 40(1) | 40(0) | 22.72±0.84 |
| 21 | 60(0) | 300(-1) | 30(0) | 30(-1) | 22.98±0.81 |
| 22 | 60(0) | 500(1) | 30(0) | 30(-1) | 24.86±0.44 |
| 23 | 60(0) | 300(-1) | 30(0) | 50(1) | 22.9±1.06 |
| 24 | 60(0) | 500(1) | 30(0) | 50(1) | 24.72±0.87 |
| 25 | 60(0) | 400(0) | 30(0) | 40(0) | 27.37±0.64 |
| 26 | 60(0) | 400(0) | 30(0) | 40(0) | 27.41±0.36 |
| 27 | 60(0) | 400(0) | 30(0) | 40(0) | 27.97±1.88 |
| 28 | 60(0) | 400(0) | 30(0) | 40(0) | 26.93±0.87 |
| 29 | 60(0) | 400(0) | 30(0) | 40(0) | 27.56±0.58 |

X_1–4_: Ethanol concentration (%), Ultrasonic power (W), Extraction time (min) and Liquid-solid ratio (mL·g^-1^).

The experimentally obtained data were fitted to a second-order polynomial equation, with all estimated regression effects listed in Table S3. The model was analyzed at a significance level of p<0.05. The linear terms (β_1_, β_2_, β_3_), the quadratic terms (β_11_, β_22_, β_33_) and the cross terms (β_12_, β_13_, β_14_) were statistically significant. After discarding non-significant terms, the model was recalculated and validated through the ANOVA test. As shown in Table 2, the model exhibited a high coefficient of determination (R^2^=0.9824). a significant regression model (p < 0.05), and a non-significant lack of fit (p > 0.05). According to Bezerra et al^[21]^, the adequacy of the model was confirmed.

Table S3. Estimated regression effects of the fitted second-order polynomial equation and ANOVA of the fitted model.

| Source | Sum of Squares | df | Mean Square | F-value | P-value | Significant |
| --- | --- | --- | --- | --- | --- | --- |
| β_0_ | 177.90 | 14 | 12.71 | 55.73 | < 0.0001 | ** |
| Linear |  |  |  |  |  |  |
| β_1_ | 1.27 | 1 | 1.27 | 5.56 | 0.0335 | * |
| β_2_ | 7.95 | 1 | 7.95 | 34.89 | < 0.0001 | ** |
| β_3_ | 3.70 | 1 | 3.70 | 16.21 | 0.0012 | ** |
| β_4_ | 0.3502 | 1 | 0.3502 | 1.54 | 0.2356 |  |
| Cross |  |  |  |  |  |  |
| β_12_ | 3.74 | 1 | 3.74 | 16.42 | 0.0012 | ** |
| β_13_ | 9.55 | 1 | 9.55 | 41.88 | < 0.0001 | ** |
| β_14_ | 1.50 | 1 | 1.50 | 6.58 | 0.0224 | * |
| β_23_ | 0.5776 | 1 | 0.5776 | 2.53 | 0.1338 |  |
| β_24_ | 0.0009 | 1 | 0.0009 | 0.0039 | 0.9508 |  |
| β_34_ | 0.5329 | 1 | 0.5329 | 2.34 | 0.1486 |  |
| Quadratic |  |  |  |  |  |  |
| β_11_ | 51.12 | 1 | 51.12 | 224.20 | < 0.0001 | ** |
| β_22_ | 85.52 | 1 | 85.52 | 375.08 | < 0.0001 | ** |
| β_33_ | 50.12 | 1 | 50.12 | 219.83 | < 0.0001 | ** |
| β_44_ | 0.0037 | 1 | 0.0037 | 0.0163 | 0.9003 |  |
| R^2^ | 0.9824 | | | | | |
| p model | 0.0000 | | | | | |
| p lack of fit | 0.2851 | | | | | |

* Significant at p < 0.05; ** significant at p < 0.01.


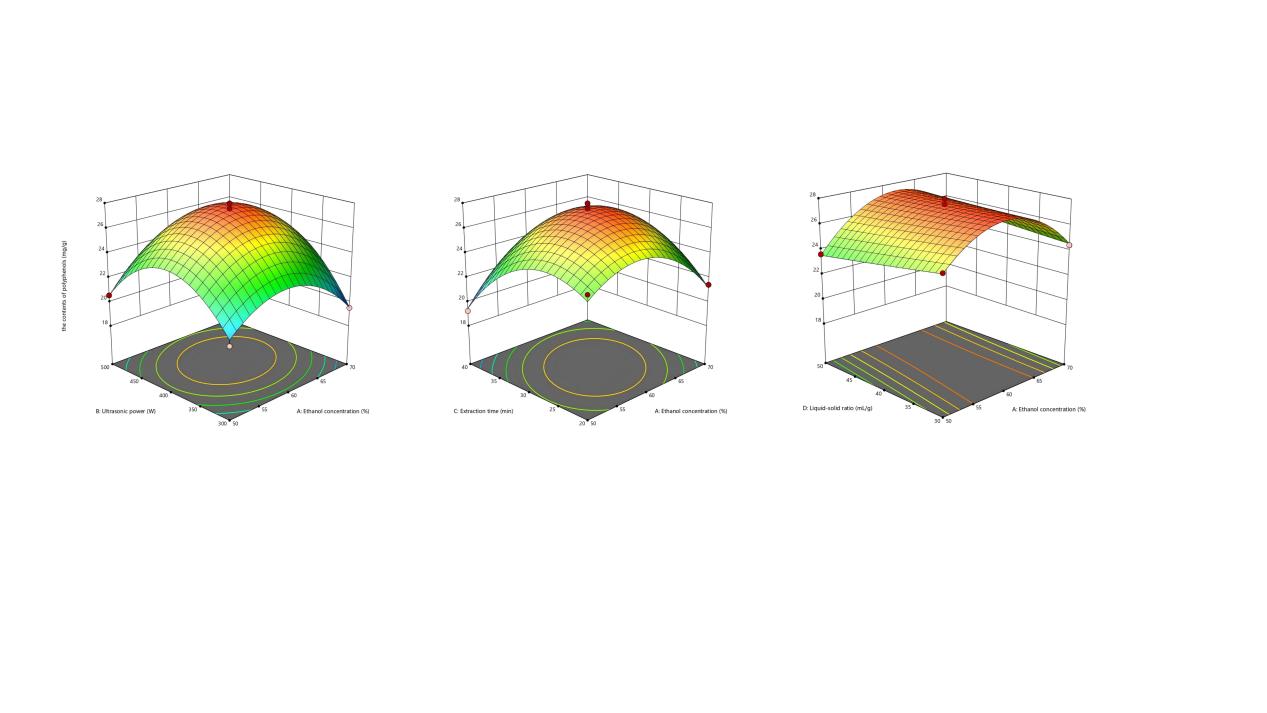


Figure S2. Response surface plots showing combined effects of process variables.

The three-dimensional surface plot presented in Figure S2 was analyzed using RSM, leading to the identification of optimal conditions. A compromise was achieved among these independent factors to set them at the smallest feasible values. The optimal conditions were determined as 60% ethanol, 410 W, 30 min, and a liquid-solid ratio of 30:1, yielding a predicted value of 27.71±0.51 (mg GAE/g E). The experimentally obtained value was 27.91±1.12, with a coefficient of variation of lower than 1% compared to the predicted value, thus confirming the validity of the model.Under these conditions, we prepared PSH-C.

**S2.2 Purification of Polyphenols**

**S2.2.1 Static Adsorption and Desorption**

As shown in Figure S3, six macroporous resins (D101, HPD100, AB-8, HPD400, HPD600, and NKA-Ⅱ) were used to purify PSH-C (1 mg/mL). The adsorption rates of HPD600 and NKA-Ⅱ were 46.78% and 50.18%, respectively, outperforming other resins. However, the desorption rate of HPD600 (80.66%) was significantly higher than that of NKA-Ⅱ (52.17%). Based on these results, HPD600 was selected for subsequent experiments.


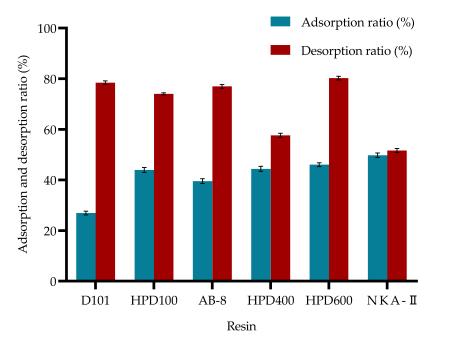
Figure S3. Adsorption and desorption capacity of resins. Error bars represent standard deviation (n = 3).

**S2.2.2 Dynamic Adsorption and Desorption**

As shown in Figure 4S (A), higher sample concentrations led to earlier leakage points, which hindered polyphenol adsorption by the resin. In this experiment, concentrations of 1 mg/mL, 2 mg/mL, and 3 mg/mL exhibited poorly defined leakage points and delayed saturation points, resulting in prolonged experimental durations and low efficiency. At concentrations of 4 mg/mL, 5 mg/mL, and 6 mg/mL, leakage points occurred at 15 mL, 10 mL, and 10 mL, respectively. However, the saturation point of the 5 mg/mL group appeared slightly later than that of the 6 mg/mL group, allowing more thorough polyphenol adsorption by the resin. Based on these findings, a sample concentration of 5 mg/mL was selected for subsequent experiments.

As shown in Figure 4S (B), the adsorption rate of polyphenols decreased with increasing sample flow rate, attributed to insufficient adsorption between polyphenols and the resin. However, lower flow rates (0.1 mL/min and 0.5 mL/min) significantly prolonged the purification time. Based on this trade-off, a sample flow rate of 1 mL/min was selected for subsequent steps.

As shown in Figure 4S (C), the desorption efficiency of perilla seed husk polyphenols varied with ethanol concentrations. Increasing ethanol concentrations produced sharper elution peaks and more concentrated polyphenol distribution in the eluent. Conversely, lower ethanol concentrations resulted in diminished peak intensity, broader peaks, and pronounced tailing. However, when the ethanol concentration reached 70%, polyphenol levels decreased significantly, likely due to reduced solubility of hydrophilic polyphenols. Based on these observations, 60% ethanol was selected as the optimal eluent.

As shown in Figure 4S (D), the desorption rate showed no significant differences at eluent flow rates ranging from 0.2–1 mL/min. However, increasing the flow rate beyond 1 mL/min resulted in a gradual decline in desorption efficiency. Therefore, 1 mL/min was chosen as the optimal eluent flow rate.

Under these conditions, we prepared PSH-P.


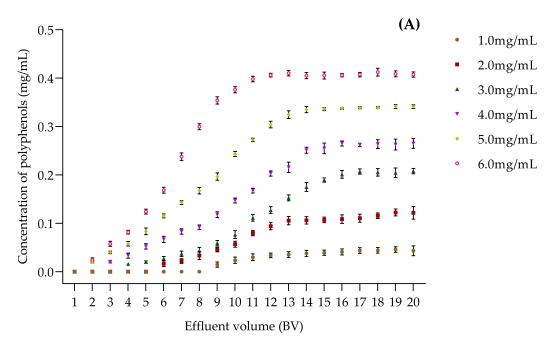

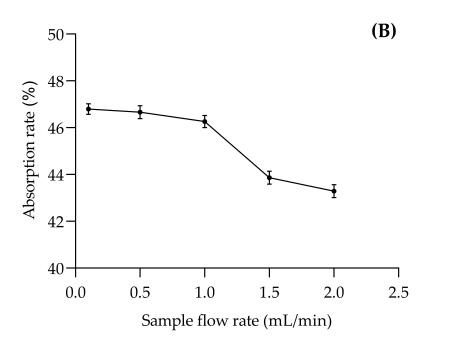


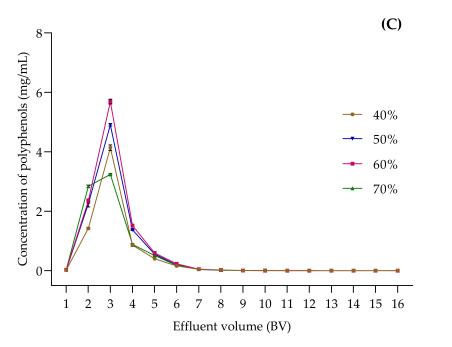

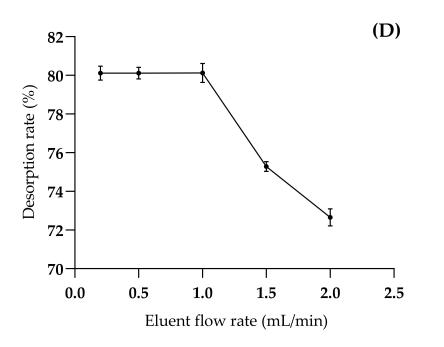


Figure 4S. Factors affecting the adsorption and desorption properties of HPD600 resin. (A) Effect of sample concentration on adsorption rate. (B) Effect of sample flow rate on adsorption rate. (C) Effect of eluente concentration on desorption rate. (C) Effect of eluente flow rate on desorption rate.Error bars represent standard deviation (n = 3).

**References**

Jin G ,Xin Z ,Chenjing M , et al.Purification of polyphenols from Phyllanthus emblica L. pomace using macroporous resins: Antioxidant activity and potential anti-Alzheimer's effects.[J].Journal of food science,2022,87(3):1244-1256.

Gao N ,Wang Y ,Jiao X , et al.Preparative Purification of Polyphenols from Aronia melanocarpa (Chokeberry) with Cellular Antioxidant and Antiproliferative Activity[J].Molecules,2018,23(1):139-139.
